# A case report on a rare case of primary tuberculous otitis media

**DOI:** 10.1016/j.joto.2023.12.003

**Published:** 2023-12-09

**Authors:** Dr Yadav Sagar Shyamlal, Dr Shikha Gianchand, Dr Rahul Kurkure

**Affiliations:** a Dept of ENT and Head Neck Surgery, 5 Air Force Hospital, Jorhat, Assam, 785005, India; b Dept of ENT and Head Neck Surgery, Armed Forces Medical College, Pune, Maharashtra, 411040, India

## Abstract

Although tuberculosis has become more common in recent years, it still accounts for just a small percentage of cases of chronic otitis media. Common symptoms of tuberculous otitis media (TOM) include otorrhoea, hearing loss, and multiple tympanic membrane perforations. Due to its rarity, the illness is frequently misdiagnosed. When a patient does not improve with standard antibacterial and antifungal treatment, this may be a possible diagnosis to explore. Complete healing is possible if the illness is diagnosed and treated timely to prevent consequences.

## 1 Background

Tuberculous otitis media (TOM) is a rare form of primary tuberculosis and classically affects the pediatric age group. The most commonly postulated mode of infection in TOM is due to bacterial ascent from the lungs, larynx, pharynx or nose. The other modes like hematogenous seeding, aspiration through Eustachian tube or direct bacterial implantation through the perforation are lesser common, but result in the characteristic features and constitutional features of primary tuberculosis (Friedland et al., 2021) (Tiwari et al., 2020) (Sebastian et al., 2020). Majority of the cases of TOM reporting to the health centres have no history suggestive of tuberculosis infection, and thus, the diagnosis of TOM is often delayed owing the rarity and indolent course of the condition. Most often complications like facial palsy, labyrinthine dysfunction, postaural fistula,etc. occur when diagnosis is late, and thus these complications bring TOM to otorhinolaryngologist's notice.

Confirmatory data on histology and microbiology are used to make the diagnosis. Since the amount of tuberculous bacilli is typically quite low upon presentation, quick diagnosis and detection by gene amplification techniques like real-time polymerase chain reaction are particularly helpful. The sooner an appropriate therapy is begun, the better is the outcome (Quaranta et al., 2011). The prognosis of uncomplicated as well as complicated cases depends on early diagnosis and treatment. Therefore, facial palsy secondary to TOM is no exception, and the prognosis for recovery depends on early treatment initiation. A shorter time interval between onset of facial palsy and initiation of anti – tuberculous treatment (ATT) leads to faster recovery. Whereas a longer time interval, as long as 2 months or more may result in no recovery at all. Among the cases of chronic otitis media, epidemiological statistics report a reduction in the incidence of TOM from 3 to 5%, “in the beginning of 20th century to 0.05–0.09% in the current era of emerging medical advances, better diagnostic modalities and implementation of Revised National Tuberculosis Control Programme (RNTCP) guidelines. Since then, the trend of TOM has only been on the decline (Sens et al., 2008).

## 2 Case report

### 2.1 History

25 year old, immunocompetent male patient with no known previous comorbidities presented to a peripheral health clinic with complaints of profuse, mucopurulent otorrhoea (R) × × 02 months. He was advised topical antibacterial drops. Having no improvement with the drops, the health care provider advised using antifungal ear drops. The patient reported no improvement with the topical antibacterial and antifungal drops. After 06 weeks of persistent otorrhoea, the patient developed a sudden (R) sided facial asymmetry. The patient was then referred to a higher health centre to consult the otolaryngologist.

## 3 On arrival to the otolaryngology clinic

### 3.1 Examination findings

The preauricular, pinna and post auricular regions in both ears were normal, no cervical lymphadenopathy was noted.

On otoscopy, external auditory canal (R) was filled with mucopurulent discharge with granulation tissue in the posterosuperior aspect of deep bony EAC. Tympanic membrane showed a large sized perforation in pars tensa. Tip of handle of malleus was visualised, no other ossicles visualised through the perforation. (Image - 1).

**IMAGE – 1:**
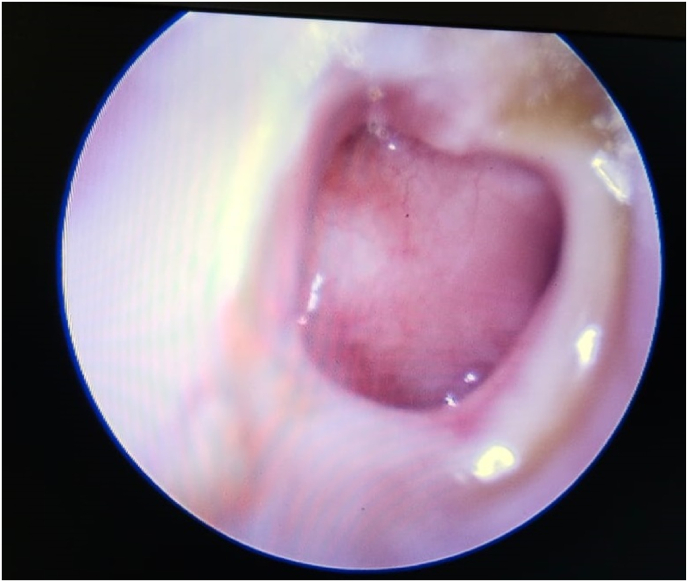
Tympanic membrane findings on arrival of the patient.

Middle ear showed soft tissue thickening.

Grade IV (House Brackmann grading system) lower motor neuron type facial nerve palsy (R) was present, there were no features of exposure keratitis seen (IMAGE- 2, IMAGE – 3, IMAGE – 4).

**IMAGE- 2:**
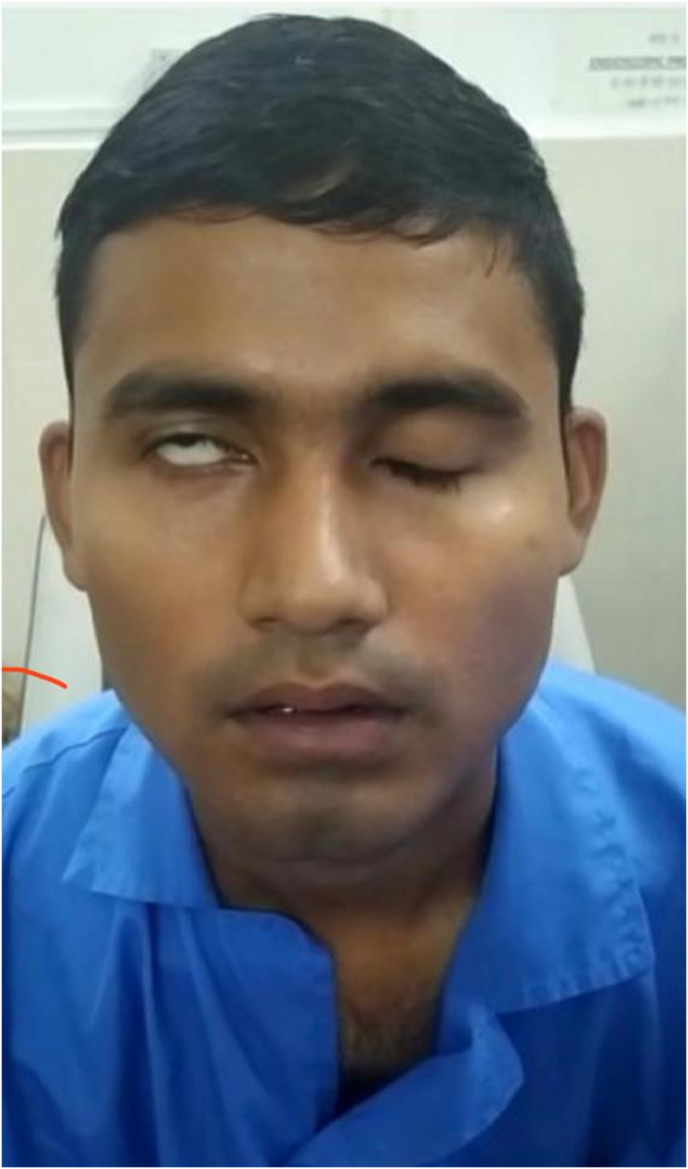
Incomplete Right eye closure.

**IMAGE – 3:**
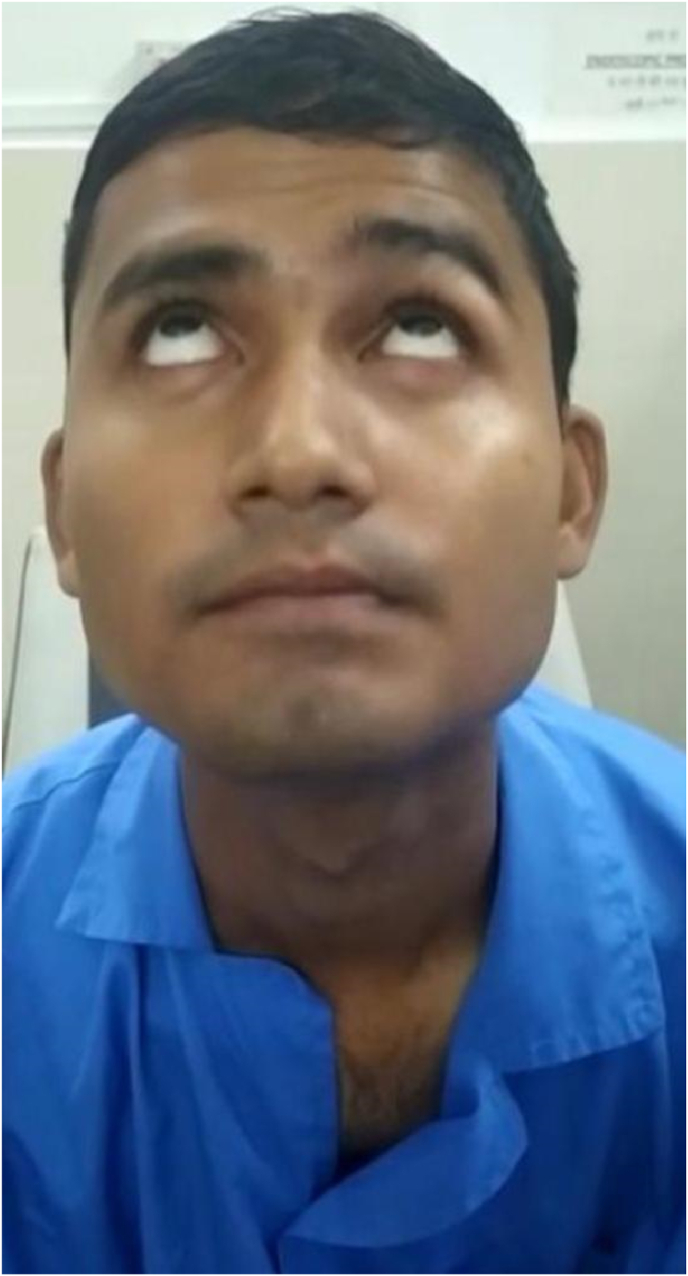
No forehead movement.

**IMAGE – 4:**
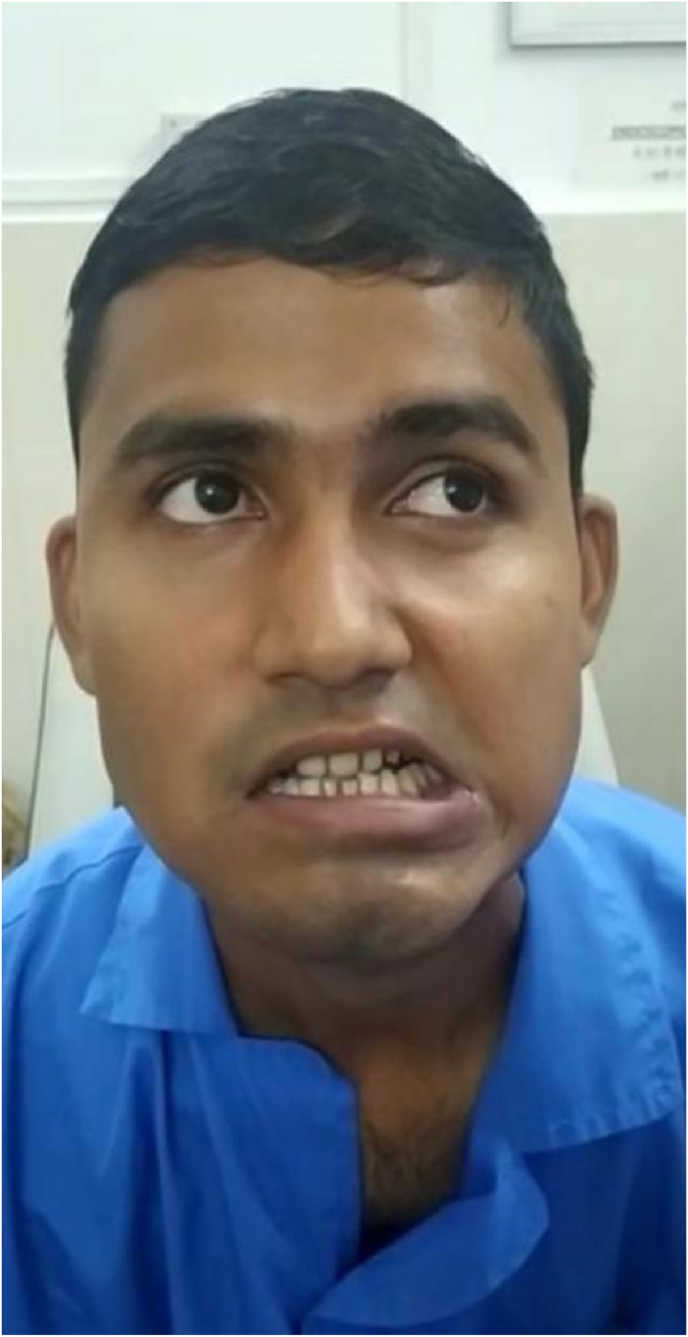
Asymmetric smile.

### 3.2 Investigations and treatment

With air conduction (AC) hearing thresholds of 65 dB HL at 250 Hz, 50 dB HL at 500 Hz, 75 dB HL at 1000 Hz, 80 dB HL at 2000 Hz, and 80 dB HL at 4000 Hz, pure tone audiometry showed substantial conductive hearing loss on the right side. On the same side, the bone conduction (BC) thresholds varied from −10 dB HL to 15 dB HL at 250, 500, 1000, 2000, and 4000 Hz. The left side had thresholds of 10, 15, 15, and 15 dB HL for AC and BC, respectively.

On a high-resolution CT scan of the temporal bone, the right middle ear and mastoid were completely opaque. Radiographic examination revealed no evidence of scutal, ossicular, or vestibular degradation (Images - 5). The tympanic section of the fallopian canal, however, was found to be dehiscent (Images - 6).

**IMAGE – 5:**
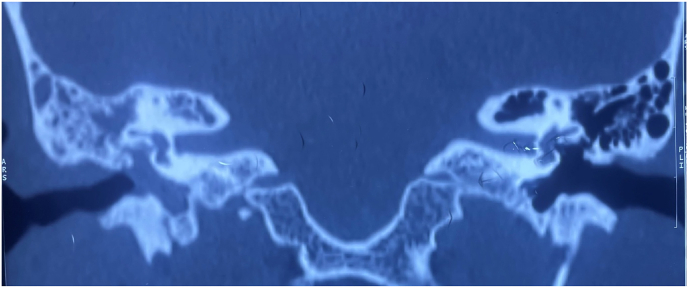
Soft tissue opacity in Right epitympanum, mesotympanum and hypotympanum (coronal view).

**IMAGE – 6:**
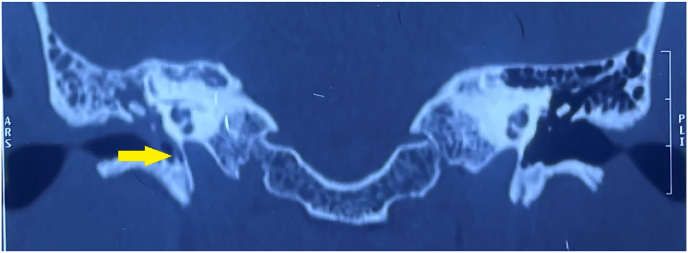
Dehiscent fallopian canal – tympanic segment (coronal view).

As a resort to reach a definitive diagnosis, Right sided tympanomastoid exploration was carried out. A cortical mastoidectomy along with type 1 tympanoplasty was done in the same sitting, Intraoperatively, pale granulations were present throughout the mastoid cavity and middle ear, extending to epitympanum and sinus tympani”. The ossicles were intact and surrounded by the granulation tissue. Diffuse pale granulations surrounded the oval window and the fallopian canal. Generalised gentle debulking of the mastoid cavity was done with utmost vigilance around the fallopian canal. The proximal segment of the fallopian canal was intact, dehiscence was noted in the distal segment. The debulked tissue taken from the middle ear and mastoid cavity was sent for microbiological and histopathological investigation.

Tuberculosis Polymerase Chain Reaction (TB-PCR) was positive and histopathological examination confirmed the presence of well – formed epitheloid granulomas and Langhans type giant cells with areas of necrosis (Images - 7,8).

**IMAGE – 7,8:**
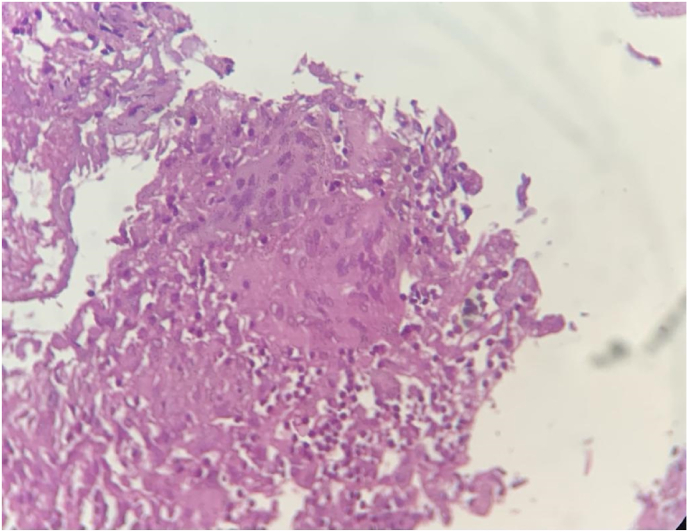

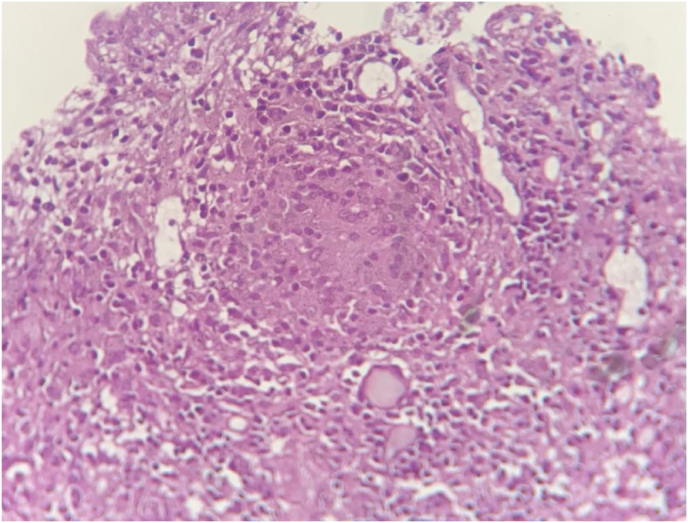
epitheloid granulomas and Langhans type giant cells with areas of necrosis

**IMAGE – 9:**
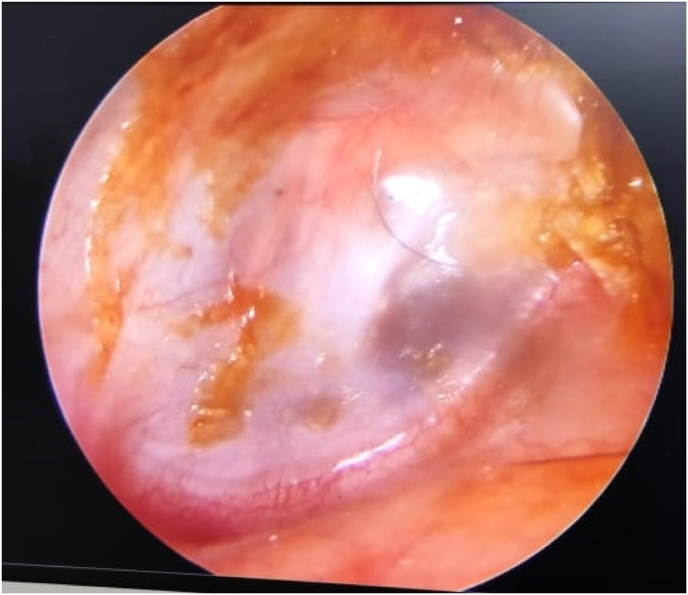
Neomemenbrane intact as seen at 6 months follow up.

On confirmation, the patient was evaluated for the focus of primary infection. Imaging of chest and abdomen revealed no abnormality.

Thereafter, patient was started on ATT as per RNTCP 2017 guidelines.

### 3.3 Follow – up

A follow up was carried out after 6 months, on conclusion of ATT. The patient showed complete resolution of symptoms. Facial asymmetry had reversed and showed complete recovery. A significant improvement in hearing was reported by the patient. The was no air-bone gap on the PTA carried out after the completion of therapy and the audiometry showed normal hearing thresholds. Besides, postaural scar had healed, the tympanic membrane was intact with no features of active or residual infection (IMAGE – 9, IMAGES – 10, 11, 12).

**IMAGES – 10, 11, 12:**
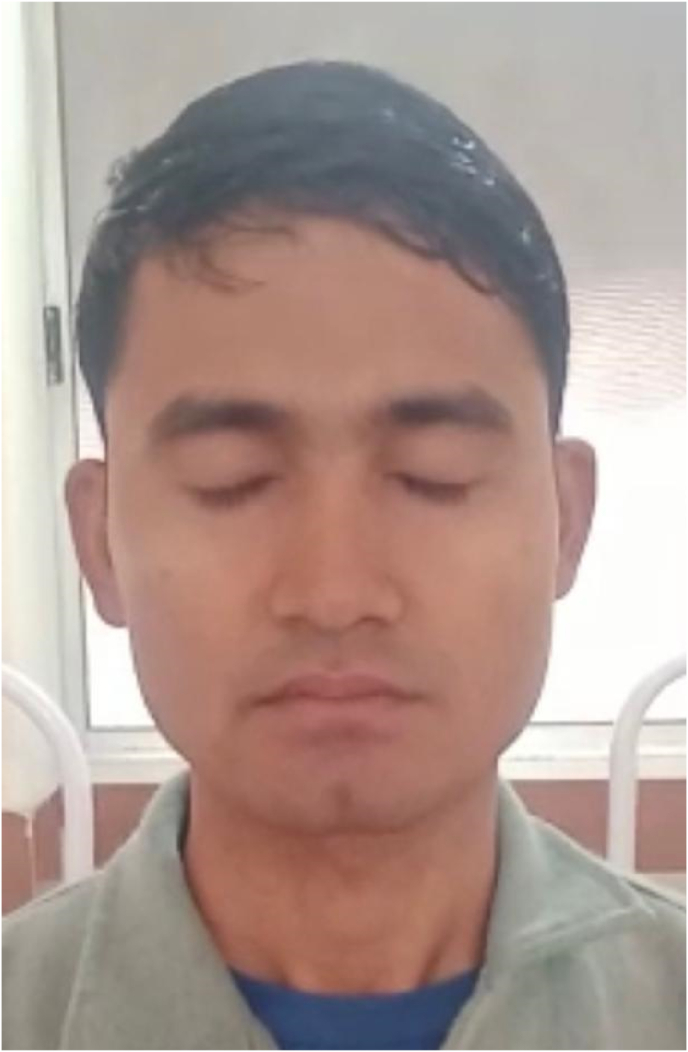

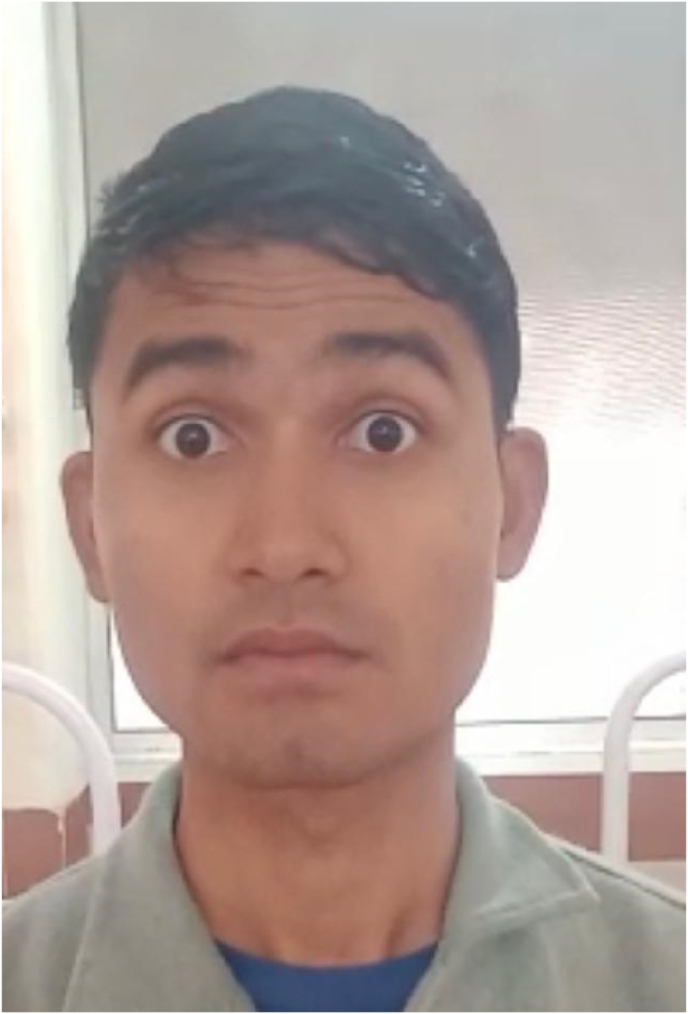

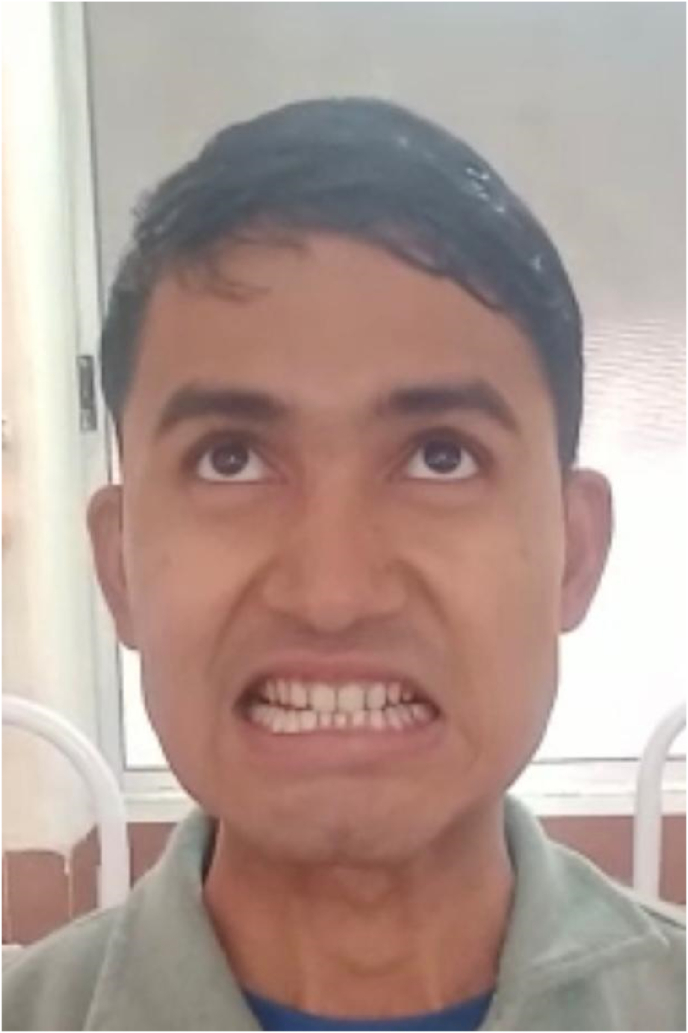
Complete facial nerve recovery with restored movements at Right forehead, eye and angle of mouth

## 4 Discussion

Only about one percent of persistent ear infections are caused by tuberculosis (Friedland et al., 2021). Infections of the ear and temporal bone may spread in one of three ways: via the bloodstream (hematogenous), directly (tympanic membrane rupture), or indirectly (Eustachian tube reflux). TOM is characterised by painless otorrhoea, numerous perforations of the tympanic membrane, and facial nerve palsy. A variety of symptoms, from complete absence to acute otalgia, a serous or purulent discharge, and sensorineural and/or conductive hearing loss, have been reported in case reports. Patient reports symptoms that are out of proportion to examination findings. One or more tympanic perforations, thickened tympanic membrane, pale granulation tissue in the middle ear and mastoid, and persistent infection are the most frequently reported examination findings (Friedland et al., 2021). The presence of ipsilateral lower motor neuron facial palsy in a case of otitis media should raise suspicion of a tuberculous aetiology, although the absence of this symptom does not rule out the diagnosis (Tiwari et al., 2020). According to the research, between 1% and 3.5% of cases which have non-tuberculous chronic otitis media also develop facial paralysis (Friedland et al., 2021).

Due to its similarity to other otological disorders, diagnosing otological TB is difficult. Due to the rarity of the disease, health care providers have a low suspicion of TOM in areas where active TB is rarely met. Due to TB being a paucibacillary illness and there being such a small amount of sample available for testing, only 20–30% of cases are microbiologically validated using ear secretion cultures. Therefore, confirmatory testing using other reliable modalities is essential (Friedland et al., 2021).

The RNTCP 2017 continues to recommend ATT as the first line of defence against tuberculosis. Definitive treatment consists of the standard Isoniazid (H), Rifampicin (R), Pyrazinamide (P) and Ethambutol (E) with the regime 2× (HRZE) plus 4× (HRE) over the course of 6 months. In the event of complications, surgical intervention is used. It includes a procedure called tympanoplasty, which cleans out the middle ear, and a procedure called mastoidectomy, which removes the mastoid reservoir. Histopathological analysis, microbial culture, or molecular testing could be used to confirm the diagnosis from a sample taken from the middle ear or mastoid cavity. Conductive hearing loss can be reversed and the condition has a very good prognosis. Auditory rehabilitation in the form of hearing aids is performed following medical treatment for sensorineural hearing loss (Thomas et al., 2022).

## 5 Lessons learnt

● Persistent otorrhoea not responding to conventional management should be viewed with suspicion.
  Differential diagnosis to be taken into consideration are recalcitrant otomycosis, malignant otitis externa, cholesteatoma, lymphoma, syphilid or other granulomatous disease.
● A high suspicion for other conditions prevents delay of definitive treatment and possible complications. Subsequently, faster and complete recovery from the complication.
● Tissue sampling aids in correct diagnosis.
● Past history of systemic disease should be elicted. However, in this case, the patient had no past history of tuberculosis nor presence of constitutional symptoms.
● TOM is highly sensitive to standard medical therapy for pulmonary tuberculosis. The treatment started promptly after confirmation of the diagnosis avoids sequelaes like incomplete facial nerve recovery, necrosis of middle ear soft tissue or erosion of ossicles and therefore, surgical interventions.
